# Comparative Performance Evaluation of Routine Malaria Diagnosis at Ho Municipal Hospital

**DOI:** 10.1155/2016/5837890

**Published:** 2016-09-25

**Authors:** James Osei-Yeboah, Gameli Kwame Norgbe, Sylvester Yao Lokpo, Mohammed Khadijah Kinansua, Loverage Nettey, Emmanuel Alote Allotey

**Affiliations:** ^1^Department of Medical Laboratory Sciences, School of Allied Health Sciences, University of Health and Allied Sciences, Ho, Ghana; ^2^School of Allied Health Sciences, University of Health and Allied Sciences, Ho, Ghana; ^3^Laboratory Department, Ho Municipal Hospital, Ghana Health Service, Ho, Volta Region, Ghana

## Abstract

Differences in quality performance score had been reported for the routinely used diagnostic methods for malaria at different settings. There is therefore a need to evaluate the test performance of the routine diagnostic methods for malaria detection in Ho, a setting with no recorded quality evaluation on malaria diagnosis. The hospital-based cross-sectional study was conducted comprising 299 outpatients. Patients were first seen and presumptively diagnosed with malaria by a clinician and were referred to the laboratory for confirmation (microscopy and Rapid Diagnostic Test). The performance analysis included sensitivity, specificity, receiver operating characteristics (ROC), weighted kappa, Youden index, and *p* value. Out of the 299 patients, 221 patients were positive by presumptive diagnosis, 35 were positive by Rapid Diagnostic Test (RDT), and 25 were positive by microscopy. Using microscopy as the gold standard, RDT had sensitivity of 62.5% and specificity of 92.73%, whilst presumptive diagnosis had a sensitivity of 70.83% and specificity of 25.82%. The RDT recorded ROC of 0.697 with *p* value of 0.0001. The presumptive diagnosis recorded ROC of 0.506 with *p* value of 0.7304. Though none of the test methods evaluated over the gold standard achieved the WHO recommended diagnostic sensitivity and specificity, the RDT achieved an acceptable agreement with the gold standard.

## 1. Introduction

Malaria continues to be a worldwide burden despite global efforts to curb the disease [1]. In Ghana, malaria is one of the main causes of adult morbidity and the leading cause of workdays loss to illness [2]. Malaria also accounts for 44% of outpatient attendance, 13% of all hospital deaths, and 22% of mortality among children less than five years of age [2]. The need for effective and practical diagnostic tests for global malaria control is increasing since effective diagnosis reduces both complications and mortality from malaria [3]. The lack of precise malaria diagnosis remains an obstacle to the treatment adherence [1]. Misdiagnosis of malaria will result in overdiagnosis, overprescription of antimalaria drugs, under diagnosis, and inappropriate treatment of nonmalaria febrile patients [4]. The World Health Organization recommends that every suspected malaria case should undergo prompt parasitological confirmation by microscopy or alternatively by Rapid Diagnostic Test [5]. Thus treatment solely on the basis of clinical suspicion should only be considered when a parasitological diagnosis is not available [5]. Clinical diagnosis of malaria is traditional among medical doctors and this method which is based on patients' signs and symptoms or on physical findings during examination is least expensive and most widely used [3]. Clinical diagnosis is widely used in areas where laboratory facilities are not available; however, it is unreliable due to the signs and symptoms of malaria being similar to other diseases [3]. Microscopy remains the gold standard for routine laboratory diagnosis of malaria, although it is not accessible and affordable in most peripheral health facilities [6]. Rapid Diagnostic Test (RDT), an immunochromatographic capture procedure, was developed to improve the timeless sensitivity and objectivity of malaria diagnosis through less reliance on expert microscopy [7]. Although RDTs clearly show promise as new diagnostic tool for Africa, it is not clear whether RDTs should replace presumptive therapy or light microscope nor is it clear which RDT is more appropriate for different epidemiological settings [8].

Despite an obvious need for improvement, malaria diagnosis is the most neglected area of malaria research [9]. Prompt and accurate diagnosis is critical to the effective management of malaria [3]. For an effective and timely treatment of malaria, the diagnostic method used should be accurate [9]. This will prevent misdiagnosis which can lead to drug misuse, increase in cost of antimalaria drugs, and also death of the patient [4]. It is estimated that a diagnostic test with 95% sensitivity and 95% specificity requiring minimal infrastructure would avert more than 100,000 deaths and about 400 million unnecessary treatments [10]. There is no known study carried out at the Ho Municipal Hospital that sought to assess the diagnostic efficiency of malaria in the facility. This study therefore seeks to comparatively evaluate the diagnosis efficiency of the various malaria diagnostic methods in the facility.

## 2. Materials and Methods

### 2.1. Study Design and Study Population

A purposive convenient cross-sectional study was carried out between January 2016 and April 2016 at the Ho Municipal Hospital in the Volta Region of Ghana. The study population is comprised of all outpatients who presented with signs and symptoms common to malaria infection (fever, bodily pains, headaches, chills, general weakness and loss of appetite, etc.). These patients, aged between five (5) months and eighty-five (85) years, were first seen, presumptively diagnosed with malaria by clinicians, and referred to the laboratory for confirmation (microscopy and Rapid Diagnostic Test). Participation was voluntary and patients who were excluded were those who were unwilling to participate, inpatients, patients reporting for review, and those with unrelated cause of ailment to malaria.

### 2.2. Sample Size Determination

Using the average monthly total malaria test requested (528) for two previous months (November 2015 and December 2015), a total study population of 2112 was generated for the four months study duration, using the Raosoft online sample size calculator (Raosoft. Inc, 2004). The recommended minimum sample of 289 participants was calculated at 95% confidence level, 5% margin of error, and a response distribution of 68% based on the average routine laboratory malaria positive test in the two previous months irrespective of the method used.

### 2.3. Blood Sample Collection

Using standard phlebotomy procedure, about 2 mL of venous blood was drawn and dispensed into ethylenediaminetetraacetic acid anticoagulant (EDTA) tubes by qualified technicians working in the hospital facility. The sample was then taken to the laboratory and used for the Rapid Diagnostic Test and field microscopy testing.

### 2.4. Rapid Diagnostic Test

Rapid Diagnostic Test was carried out as routinely done without any special attention given to the samples by biomedical scientist working in the municipal hospital laboratory. All Rapid Diagnostic Tests were done using Bioline SD malaria antigen Pf. manufactured by Standard Diagnostic, Inc, Korea. Assays were carried out as described by the manufacturer. In brief, all kit components and specimen were brought to room temperature prior to testing. Using a 5 *μ*l disposable capillary pipette, whole blood was drawn and transferred into the round sample well. Four (4) drops of assay diluent was added into the square assay diluent well holding the diluent bottle vertically. Reading of test was done in 15 minutes and for samples that tested negative repeated reading was done in 30 minutes. The presence of one colour band (“C” Control line) within the result window was interpreted as negative results. The presence of two colour bands (“T” Test line and “C” Control line) within the result window was interpreted as positive results. In the event where the control line fails to appear within the results window, the test was invalidated.

### 2.5. Microscopy

An amount of 6 *μ*L and 2 *μ*L of the blood sample was pipetted for the preparation of thick and thin blood films, respectively. Thin film was fixed with methanol for 5 minutes and both thin and thick film were stained with 10% Giemsa for 10 minutes. Stained slides were left to air-dry before examination using a 100x objective oil immersion light microscope. The smears were independently read by two microscopists who were blinded to the results of the RDT as well as the diagnosis made by the clinicians and between each other. Parasites were counted against 200 white blood cells (WBCs) from the thick film. The parasite density was obtained by assuming a total WBC count of 8000/mL and at least 200 fields were examined before being taken as a negative result.

### 2.6. Data Analysis

The sensitivity and specificity of each of the three test methods were calculated by comparing to a composite reference gold standard generated from the three methods. The composite reference method was defined as a method that was positive for malaria parasites by all of the three methods (Presumptive, RDT, and Microscopy) and also negative for malaria parasites by all of the three methods. This gives the method 100% hypothetical sensitivity, specificity, and positive and negative predictive values [7].

Taking blood slide microscopy as the gold standard, the performance of the presumptive diagnosis method and the Rapid Diagnostic Test was evaluated to generate diagnostic accuracy summary statistics including receiver operative characteristics test, weighted kappa, *p* values, and Youden J. statistics using MedCalc Version 14.2.0.0 for Windows (Vienna, Austria, https://www.medcalc.be/).

## 3. Results

Using a composite reference as gold standard which was generated from the three diagnostic methods, only 10 people were found to be truly positive for malaria and 65 people were found to be truly negative giving a hypothetical 100% sensitivity and specificity. Evaluating each of the diagnostic methods over the composite reference, RDT reported a sensitivity of 4.78% and a specificity of 72%. The Presumptive diagnosis method showed a sensitivity of 43.48% and a specificity of 23.55%, while the field microscopy method had a sensitivity of 4.55% and a specificity of 82.28%. Using field microscopy as the gold standard, RDT reported a sensitivity of 62.5% and a specificity of 92.73%, while presumptive diagnosis reported a sensitivity of 70.83% and a specificity of 25.82%. The presumptive diagnosis was found to have a low positive predictive value (7.69) against the field microscopy technique. See Table 1.

Comparing the two diagnostic methods to field microscopy in relation to the different age groups, RDT reported the highest sensitivity of 85.71% among ≤5 years age group and specificity of 100% among the age group of 6–18 years. Using presumptive diagnosis, the highest sensitivity of 75% was reported among the age group of 19–40 years and a specificity of 92.31% was reported for the 6–18 years age bracket. The presumptive diagnosis exhibited a low positive predictive value across the different age categorization; the exception though was found for the 6 to 18 years group. See Table 2.

The quality of the test method as a medical diagnostic tool was assessed using the Youden J. index on a scale of 0-1 as shown in Tables 3 and 4. The performances of the RDT and presumptive diagnosis evaluated using field microscopy as the gold standard reported Youden J. index of 0.395 and 0.013, respectively. The gender stratified performance evaluation revealed higher diagnostic ability for RDT method compared to the presumptive diagnosis method irrespective of the gender categorization.

Using the interrater agreement analysis, the weighted kappa was estimated to assess how results produced by RDT and presumptive diagnosis agree with field microscopy in the diagnosis of malaria. Agreement ranged from moderate (0.457) for RDT to poor (−0.007) for presumptive diagnosis.

Assessing the effectiveness of the RDT and the presumptive diagnosis as methods for the diagnosis of malaria, the receiver operating characteristic (ROC) analysis was used to estimate the area under the diagnostic curve (AUC), ranging from 0.5, a worthless test, to 1, a perfect test. The RDT was shown to be a more effective test method (AUC = 0.697; *p* < 0.0001) than the presumptive diagnosis (AUC = 0.506; *p* = 0.7304). As seen in Tables 3 and 4, gender variations in performance were not prominent for both diagnostic methods (RDT and presumptive diagnosis). See Table 3.

Out of the 24 patients that were reported as having positive malaria parasite by the field microscopy method, 15 and 17 were positive for RDT and presumptive diagnosis, respectively (true positive). Among the 264 patients who tested negative for RDT, 9 were positive for field microscopy (false negatives). Among the 78 patients reported to be negative by presumptive diagnosis, 7 tested positive using the field microscopy technique (false negative). Among the false negative recording using the RDT 11.11% was within a parasitic count of 101–1000/*μ*l and the rest (88.89%) had a parasitic count of 1000/*μ*l or greater. Among the false negative recording using presumptive diagnosis 14.29% was within the parasitic density of 1–100/*μ*l and the rest (85.71%) presented with parasitic density of 1000/*μ*l and above. See Table 4.

## 4. Discussion

Malaria is a deadly disease especially in children and, for that reason, it is important to have a prompt and accurate diagnosis of the disease [11]. In this study, the test performance of the three routine malaria diagnostic methods, namely, Rapid Diagnostic Test (RDT), presumptive diagnosis method, and field microscopy technique, was evaluated. The diagnostic accuracy of these methods was measured against a composite and field microscopy as gold standards. In general, the study established that the sensitivity of the three diagnostic methods was very low when evaluated over the composite reference. This is an indication of wider differences or substantial nonoverlap between the test methods in the positive detection of malaria among the study population [6]. In contrast, the sensitivities recorded by the individual test methods were lower than that reported in a similar study in Nigeria by Ojurongbe et al. [7] which had 62.3% and 77.2% for RDT and field microscopy, respectively. This difference in recorded sensitivity could be accounted for by the makeup of the composite reference where polymerase chain reaction was inclusive in the previous study but this is not included in this study. Again, whereas this study made use of presumptive diagnosis, the study by Ojurongbe et al. [7] did not. Thus, problems arise in comparison when established methods are used as a composite gold standard without allowing for inherent accuracies for the individual test methods [12].

Diagnosis of malaria has traditionally been based on presumptive diagnosis [4] and its principles are based on nonspecific signs and symptoms like headaches, fever, weakness, dizziness, vomiting, abdominal pains, myalgia, chills, and pruritus [7]. In the current study presumptive diagnosis demonstrated a generally low specificity when compared with the two gold standards: composite (23.55%) and field microscopy (25.82%) as shown in Tables 1 and 2, respectively. These findings were consistent with a previous work which recorded a specificity ranging from 0 to 9% [13]. Thus, presumptive diagnosis is unable to identify patients without malaria as truly uninfected (false positives). This leads to treating all fevers presumptively as malaria, thereby masking underlying potentially fatal conditions [14]. From the present study, out of 221 people who were clinically diagnosed with malaria, 17 and 24 were positive by field microscopy and RDT, respectively. This shows that about 70% of patients who were diagnosed as having malaria presumptively turned out to be parasite negative. This massive malaria overdiagnosis according to Reyburn et al. [15] threatens the sustainability of deployment of artemisinin combination treatment, and treatable bacterial diseases are likely to be missed.

After age stratification, the highest sensitivity for the two test methods against the field microscopy technique was recorded among ≤5 years group (Table 2). This agrees with the findings of Nkrumah et al. [16] where an increase in the sensitivity of a* histidine-rich protein 2* (Pfhrp2) assay was found in children compared to adults. Nkrumah et al. [16] posited that lower immunity and possibly less interference by antibodies among children could be attributable to such outcome. Parasitaemia in older aged groups often remains very low and frequently undetectable by conventional malaria diagnostics (microscopy) and Rapid Diagnostic Test [17].

Rapid Diagnostic Tests (RDTs) based on* histidine-rich protein 2* (Pfhrp2) have shown a varied accuracy for malaria infection in field studies, with field microscopy taken as the gold standard [1, 18–20]. In the present study, RDT reported a sensitivity of 62.5% and specificity of 92.93% when compared with field microscopy. This is within the same range as previous studies [21, 22]. However, the sensitivity was lower than the World Health Organization (WHO) recommended minimal standard of 95% sensitivity for* Plasmodium falciparum* densities of 100/*μ*L and a specificity of 95% for an acceptable Rapid Diagnostic Test for malaria [23]. The diagnostic accuracy of RDTs can be affected by several factors such as quality of the products, storage temperature, humidity, and end users' performance [6].

Persistence of Pfhrp2 protein in circulation after parasite clearance contributes to lower specificity level of RDT [9, 19, 21]. This may explain the observation in the current study where 9 participants on malaria treatment tested positive for RDT with no parasitic detection by field microscopy (false positives). In the case where the parasite density is below the threshold for detection by microscopy (submicroscopic parasitic density), it is still possible for RDT to report positive malaria test [19] or low density parasitaemia might be missed and wrongly classified as negative with microscopy; however the double blind reading of blood films by two experienced microscopists was aimed at reducing the latter scenario [24].

In the current study, out of the 264 patients that RDT confirmed as negative, field microscopy detected 9 of them as positive with 8 patients recording a parasitic density of ≥1000 parasites/*μ*L (Table 4). Studies have reported patients with high levels of parasitaemia that give false negative RDT results due to the deletion of Pfhrp2 antigens or genetic variability in the Pfhrp2 gene in certain* Plasmodium falciparum* parasites [25, 26].

In a study by Abeku et al. [24], false positive error rates declined with increasing age of patients and this was probably a result of acquired immunity in clearing parasite antigen. Contrary to this, the present study noted false positive results by RDT increased with increasing age of patients. According to the manufacturer's manual, internal evaluation was performed on the SD Bioline malaria Ag Pf. test which reported a 100% sensitivity for parasitic count between 101 and 500 parasites/*μ*L but the present study reported a sensitivity of 75% for a similar parasitic count of 101–1000 parasites/*μ*L. Manufacturer's specified sensitivity was 100% for a parasitic count greater than 1000 parasites/*μ*L whilst this study had a sensitivity of 57.89% for that same parasitic count. The manufacturer's overall sensitivity when microscopy was used as a reference was greater or equal to 99% whilst this study reported 62.5% overall sensitivity.

A patient who reported* Plasmodium malariae* infection was also positive with the RDT which has been designed to detect only* Plasmodium falciparum*. This revelation may be due to cross-reactivity [9]. RDT also was positive to a parasite count that was as low as 40 parasites/*μ*L and this is in contrast to other studies [6, 10, 27].

The weighted kappa statistic, widely used as a chance-corrected measure for nominal agreement [28], was used to test interrater agreement between microscopy and the other two diagnostic methods (RDT and presumptive diagnosis). There was a moderate level of agreement between RDT (kappa: 0.457) and microscopy. This observation is in accordance with the findings of Ali et al. [29] who reported a moderate agreement between RDT and field microscopy in Cameroon. But the agreement observed was slightly higher compared to that reported by Kilonzo et al. [30] (kappa: 0.354) in Tanzania. A recent study in Yemen by Alareqi et al. [31] also reported a kappa of 0.379 for both febrile and afebrile participants and a higher weighted kappa of 0.638 among only febrile patients. Thus RDT could be used for malaria diagnosis in settings where microscopy is not available [32]. Presumptive diagnosis (kappa: 0.007) however had a poor or no agreement with microscopy and thus it is likely to misdiagnose malaria if it is not used alongside a laboratory based diagnostic tool.

The discriminating power of RDT and presumptive diagnosis for the detection of malaria in patients was further investigated by the area under the receiver operative characteristics (ROC) as seen in Table 3. It was found that RDT (area under the diagnostic curve (AUC: 0.697)) was more effective in predicting malaria infection; however in the work of Djimde et al. [33] in Mali, RDT recorded a higher area under the ROC curve (0.97). The presumptive diagnosis method had an area under the ROC curve of −0.506, which implies that it is a worthless test for malaria diagnosis.

Youden index was carried out to measure the medical usefulness of the diagnostic methods for malaria detection. RDT proved to be a useful diagnostic test (Youden: 0.395) as compared to presumptive diagnosis (Youden: 0.013). The performance of the RDT agrees with the findings of Samadoulougou et al. [34] in Burkina Faso who reported an overall Youden of 0.40. This shows that presumptive diagnosis is less effective and an unreliable diagnostic tool for malaria diagnosis as a standalone test.

## 5. Conclusion

Based on the results of this study, none of the test methods evaluated over the gold standard (field microscopy technique) achieved the WHO recommended diagnostic sensitivity and specificity. However, the RDT achieved an acceptable agreement with the gold standard. Factors such as medication and age also played a role in influencing the test performance of the various diagnostic methods. Diagnosis of malaria by field microscopy should still be the gold standard although it requires a level of expertise. In settings where microscopy is not available, however, RDTs must be preferred as a confirmation of presumptive diagnosis.

## Figures and Tables

**Table 1:** Performance of rapid, presumptive, and microscopic diagnostic test using the composite and microscopy as gold standards.

Parameter	Sensitivity	Specificity	PPV	NPV
*Composite as a standard*				
Rapid Diagnostic Test	4.78	72.22	28.57	24.62
Presumptive diagnosis	43.48	23.55	4.52	83.33
Field microscopy	4.55	82.28	41.67	23.64
*Microscopy as a standard*				
Rapid Diagnostic Test	62.50	92.73	42.86	96.59
Presumptive diagnosis	70.83	25.82	7.69	91.03

Data is presented as percentages. PPV: positive predictive value and NPV: negative predictive value.

**Table 2:** Age stratified performance of rapid and presumptive diagnostic test using field microscopy as a gold standard.

Parameter	Sensitivity	Specificity	PPV	NPV
*≤5 years*				
Rapid Diagnostic Test	85.71	86.89	42.86	98.15
Presumptive diagnosis	71.43	26.23	10.00	88.89
*6–18 years*				
Rapid Diagnostic Test	30	100	100	85.11
Presumptive diagnosis	7.41	92.31	66.67	32.43
*19–40 years*				
Rapid Diagnostic Test	37.5	96.88	50.00	94.90
Presumptive diagnosis	75	30.21	8.22	93.55
*41–85 years*				
Rapid Diagnostic Test	50	97.18	60.00	95.83
Presumptive diagnosis	66.67	22.95	7.84	87.50

Data is presented as percentages. PPV: positive predictive value and NPV: negative predictive value.

**Table 3:** Interrater diagnostic performance criteria for rapid and presumptive diagnosis of malaria with field microscopy as a gold standard.

Parameter	AUC (ROC)	*p* value	Kappa	Youden
*Total population*				
Rapid Diagnostic Test	0.697	<0.0001	0.457	0.395
Presumptive diagnosis	0.506	0.7304	−0.007	0.013
*Male participants*				
Rapid Diagnostic Test	0.702	0.0035	0.492	0.404
Presumptive diagnosis	0.504	0.8891	0.005	0.009
*Female participants*				
Rapid Diagnostic Test	0.695	0.0005	0.436	0.390
Presumptive diagnosis	0.512	0.6156	−0.013	0.023

AUC: area under curve and ROC: receiver operative characteristic. Kappa <0.20: poor, 0.41–0.60: moderate, 0.61–0.80: good, and 0.81–1: very good. *p* is significant at 0.05.

**Table 4:** Dose response threshold for rapid and presumptive diagnostic tests stratification by parasite density in thick blood smear.

Parameter	Parasitic count (count/*μ*l)		
	1–100	101–1000	≥1000
*Field microscopy*			
Positive	**1**	**4**	**19**
*Rapid Diagnostic Test*			
Positive	1 (100)	3 (75.00)	11 (57.89)
Negative	0 (0.00)	1 (25.00)	8 (42.11)
*Presumptive diagnosis*			
Positive	0 (0.00)	4 (100)	13 (68.42)
Negative	1 (100)	0 (0.00)	6 (31.58)

Data is presented as figures and percentages.
